# High-throughput assessment of mechanical properties of stem cell derived red blood cells, toward cellular downstream processing

**DOI:** 10.1038/s41598-017-14958-w

**Published:** 2017-10-31

**Authors:** Ewa Guzniczak, Maryam Mohammad Zadeh, Fiona Dempsey, Melanie Jimenez, Henry Bock, Graeme Whyte, Nicholas Willoughby, Helen Bridle

**Affiliations:** 1 Heriot-Watt University, School of Engineering and Physical Science, Department of Biological Chemistry, Biophysics and Bioengineering Edinburgh Campus, Edinburgh, EH14 4AS Scotland; 2 MedAnnex Ltd, 1 Summerhall Place, Techcube 3.5, Edinburgh, EH9 1PL Scotland; 3 University of Glasgow, School of Engineering, Biomedical Engineering Division, Glasgow, G12 8QQ Scotland

## Abstract

Stem cell products, including manufactured red blood cells, require efficient sorting and purification methods to remove components potentially harmful for clinical application. However, standard approaches for cellular downstream processing rely on the use of specific and expensive labels (*e.g*. FACS or MACS). Techniques relying on inherent mechanical and physical properties of cells offer high-throughput scalable alternatives but knowledge of the mechanical phenotype is required. Here, we characterized for the first time deformability and size changes in CD34+ cells, and expelled nuclei, during their differentiation process into red blood cells at days 11, 14, 18 and 21, using Real-Time Deformability Cytometry (RT-DC) and Atomic Force Microscopy (AFM). We found significant differences (p < 0.0001; standardised mixed model) between the deformability of nucleated and enucleated cells, while they remain within the same size range. Expelled nuclei are smaller thus could be removed by size-based separation. An average Young’s elastic modulus was measured for nucleated cells, enucleated cells and nuclei (day 14) of 1.04 ± 0.47 kPa, 0.53 ± 0.12 kPa and 7.06 ± 4.07 kPa respectively. Our identification and quantification of significant differences (p < 0.0001; ANOVA) in CD34+ cells mechanical properties throughout the differentiation process could enable development of new routes for purification of manufactured red blood cells.

## Introduction

Annually, around 112.5 million blood donations are collected across 176 countries in over 13000 blood centres^1^. However, each year the World Health Organisation reports a shortage of safe donated blood for transfusion, mostly due to a decreasing number of people eligible to donate blood and technical limitations for blood long-term storage^2^. There is consequently an urgent need for easily manageable alternative sources of red blood cells (RBCs), one option being their manufacture from stem cells^3–6^. Manufactured red blood cells (mRBCs) have been positively validated as a potential clinical product in 2011 by Giarratana *et al*.^7^, with potentially transfusable RBCs already produced *in vitro* using embryonic stem cells^5^, induced pluripotent stem cells^8^, CD34+ cells sourced from bone marrow^4^ or umbilical cord blood^9^, and recently an immortalized adult human erythroid line (Bristol Erythroid Line Adult BEL-A)^10^. However, the differentiation protocols currently used are not 100% efficient. Specifically, the end-product of the protocol is a heterogeneous cell mixture containing not only fully functional enucleated mRBCs, but also free-floating nuclei expelled during enucleation and undifferentiated nucleated cells. The presence of residual stem cells, partially differentiated cells and nuclei, pose a health risk if injected into patients^11^ and is consequently a serious concern that must be alleviated for mRBCs and other cellular therapies by developing adequate purification procedures^12–17^. Traditionally, target cell separation is performed by Fluorescent Activated Cell Sorting (FACS) and Magnetic Activated Cell Sorting (MACS). Both techniques are very specific since they utilize molecular biomarkers but require the addition of costly modifying agents, such as antibodies or DNA stains, and separate quality-control processes^18,19^. Furthermore, the throughput of these techniques is limited (*e.g*. 10^7^ cells/ hour for the FACS instrument used in this research) and industrially viable technologies require cost-effective, automated and scalable approaches^20^. Various label-free approaches have been proposed in the literature, though have not yet reached the stage of commercial testing: acoustophoresis^21^, magnetophoresis^22^, optical methods^23^ and passive sorting^24^. Passive sorting (such as inertial focusing^25^, pinch flow fractionation^26^, deterministic lateral displacement^27^ and filtration^28^) exploits properties of the device design and, except for a liquid pumping system, they do not require any other external forces. These systems present many potential advantages such as a reduced number of sample processing steps (*e.g*. by reducing staining/washing steps), relatively high-throughput (millilitres/min^29^ and litres/min^30^) and efficiency (>90%)^31–33^. However, sorting in this type of system is facilitated purely by endogenous cell properties such as size and deformability and further evidence is needed to quantify the cell mechanotype, to overcome the existing lack of knowledge on e.g. mRBCs mechanical properties^34^, and determine the potential for mechanotype based sorting.. Deformability is emerging as a novel homogeneity marker that could serve to identify subpopulations within complex cell samples such as mRBCs^35^. However, while qualitative observations have noted changes in phenotype throughout the differentiation protocol of CD34+ cells, little is known about their mechanical phenotype changes. This article reports on the first extensive quantitative analysis of these changes, combining both high-throughput microfluidics as well as traditional biophysical characterisation. Specifically, we characterised for the first time the mechanotype of cord blood CD34+ undergoing *in vitro* differentiation into RBCs, focusing on four key stages. Data was collected determining the size and deformability of enucleated cells, nucleated cells and free-floating nuclei using real-time deformability cytometry (RT-DC), atomic force microscopy (AFM), and bright field/fluorescent imaging. Furthermore, staining of the nucleus and cytoskeletal proteins was undertaken to investigate the potential contribution of these factors to the observed mechanotypical changes.

## Results and Discussion

The *in vitro* manufacture of RBCs from hematopoietic stem cells (CD34+) follows an *in vitro* protocol which is a recapitulation of *in vivo* erythropoiesis through distinct developmental stages^36,37^ (for details of the protocol and the different stages involved consult Fig. S1). Initially, the culture is expanded for the first ten days (D0 to D10) before differentiation is induced at D11, resulting in drastic cell phenotype changes during the final 11 days of differentiation. Observed changes are induced stage-wise, by adjusting cell culture medium components. The presence of biological markers at different points in the differentiation has been studied^8,9,38^, underpinning the label-based separation approaches, and it is known that between D0 and D11, CD34+ cells extensively proliferate without changing their identity. Around D14 cells start producing haemoglobin and reduce their intracellular structures (the cytoplasm becomes simplified) and size. By D18, chromatin becomes compacted, cellular division slows and in the final stages, the nucleus is expelled. Based upon this, four distinct time points (at D11, D14, D18 and D21) were selected to assess the changing mechanotype of CD34+ during *in vitro* erythropoiesis to determine the potential for mechanical properties to act as a homogeneity marker upon which passive cell separation methods can be developed.

### High-throughput size and deformability assessment

While there are many available well-established technologies for assessing cell mechanotype such as Atomic Force Microscopy (AFM)^39^, micropipette aspiration^40^, magnetic tweezers and optical stretchers^41^, these methods suffer from low-throughput^42^. To assess a high number of cells (thousands of events per minute), we used a microfluidic-based Real-Time Deformability Cytometer (RT-DC)^43^. RT-DC is a contactless technique, allowing gain of thousands of events per minute, which is convenient for the global characterisation of complex samples^44^. For comparison of technologies for cell mechanotype assessment see Table S1.1. In the RT-DC set-up, shear stress is generated by a viscous liquid flowing through a channel of defined dimensions to induce cell deformation, which is defined as cell circularity^45^ and is given by:

1 $$c=2\sqrt{\pi A}/l$$

where $A$ is the projected cell surface area and $\,l$ is the cell perimeter. For perfectly circular objects $c=1$ and a deformable object will be characterised by $c < 1$.

**Figure 1:**
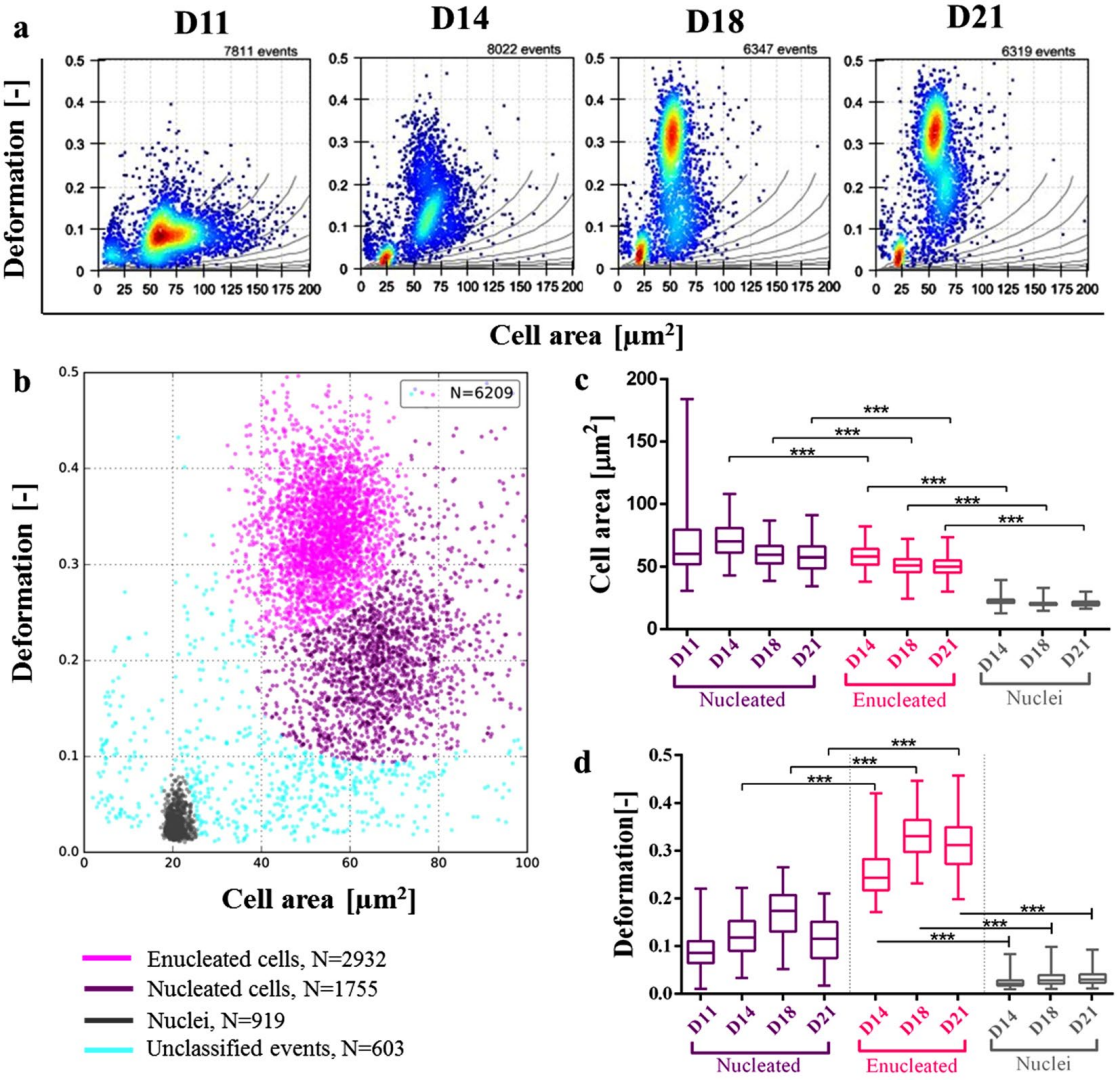
**(a)** Scattered plots obtained from RT-DC for CD34+ undergoing *in vitro* haematopoiesis corresponding to four time points: D11, D14, D18 and D21. Cells are flowing at 0.12 µl/min through a 20 µm × 20 µm channel. Each dot represents a single event (the total number of collected events is displayed on the top of each diagram). Colours indicate a density scale. Grey isoelasticity lines on the scatter plots represent a predicted cell deformability for cells of the same elasticity and different size^45^ **(b)** By analysing raw data with a Gaussian mixture model at least three subpopulations within sample from D18 were detected and colour-coded, corresponding to nucleated (purple), enucleated cells (pink) and nuclei (grey). Remaining events (blue) are considered unclassified events, artefacts and cell debris. **(c,d)** Box plots summarizing cell area and deformation respectively. Values for each subpopulation are extracted from raw data by gating enucleated, nucleated cells and nuclei as justified in Fig. S2. P-values were calculated using a generalized mixed model (***p < 0.0001). The line in the box represents the median and the box itself represents data from lower and upper quartile while the whiskers correspond to the lowest and highest extreme values.

CD34+ cells size and deformability at D11, D14, D18 and D21 were assessed using RT-DC for both individual and mixed populations of nucleated and enucleated cells as well as expelled nuclei. Free-floating nuclei and undifferentiated cells constitute the two main contaminants that must be removed from the sample, prior to clinical application, to leave purified mRBCs. A global view of mechanical changes using mixed populations, directly from the cell culture, is presented in Fig. 1a, plotting deformation against cell area. The results indicate that as the differentiation process progresses, deformability increases while size decreases and the emergence of a greater number of different cell populations can be observed. At D11 cells are strongly heterogeneous in size (cell areas ranging between 25 and 175 µm^2^) while deformability is low (<0.2). At D14 the first spontaneous enucleation events are observed characterized by a new emerging subpopulation on the deformation axis (events with deformation >0.2) and the reduction in cell size very few cell areas above 100 µm^2^). Closer to the end of the differentiation process (D18 and D21), the enucleated cell subpopulation becomes dominant (deformability >0.25) and another population corresponding to expelled nuclei appears (deformability < 0.03). In *in vivo* differentiation, nuclei would be removed by macrophages^46,47^.

Utilising the above data, we compared the size and deformability of enucleated and nucleated cells and nuclei at each of the time points. Firstly, we confirmed the identity of the three subpopulations by collecting data from a mixed sample as well as from samples which were sorted into pure populations by FACS and assessed using the RT-DC separately (Fig. S2). Regions corresponding to each subpopulation were therefore identified and used for analysis (using the polygon tool in the ShapeOut software) (Fig. S2).

The populations identified from the mixed sample (Fig. S2a) correspond to the individual gated enucleated (Fig. S2d) and nucleated (Fig. S2e) cells and nuclei (Fig. S2c) populations. In general, the individual populations of cells are slightly stiffer than those from the mixed population, due to the extra processing undertaken^48^.However, Fig. S2b shows that the three different subpopulations plot in different regions suggesting that there is potential to utilise size and deformability changes as a basis for separation and sorting. Secondly, to confirm that software could utilise size and deformability differences to classify cells, events from the scatter plot of a mixed sample were analysed using a Gaussian mixture model also identifying three subpopulations (Fig. 1b), as expected from the previous gating analysis (Fig. S2).

To further analyse the data, box plots were generated showing the average and range of the size and deformability characteristics for each of the subpopulations. Figure 1c illustrates the size variation of the three populations at the selected time points (D11 only appears for nucleated cells as this is before the enucleation step). Nuclei are much smaller (with area 22.8 ± 3.8 µm^2^, mean ± SD) than the nucleated cells (D11 72.9 ± 25.8, D14 71.6 ± 12.8, D18 54.7 ± 7.2 and D21 52. 2 ± 7.5 µm^2^) and enucleated cells (D14 57.6 ± 8.6, D18 50.9 ± 9 and D21 47.9 ± 7.5 µm^2^) with little size overlap (p < 0.0001). In terms of the size of cells, there is a statistically significant difference between enucleated and nucleated cells (p < 0.0001). However, there is an overlap between events from both subpopulations (Fig. 1c).

Figure 1 d considers the deformability differences between the three populations and demonstrates greater discrimination potential. Nuclei deformability is 0.03 ± 0.01 whereas that of nucleated cells is greater than 0.1 (D14 0.13 ± 0.04, D18 0.18 ± 0.05 and D21 0.12 ± 0.05) and enucleated cells greater than 0.25 (D14 0.26 ± 0.05, D18 0.34 ± 0.05 and D21 0.33 ± 0.05) and the difference is statistically significant (p < 0.0001). Additionally, a significant difference in deformability (p < 0.0001) is observed for nucleated (deformability 0.1–0.25) and enucleated (deformability >0.25) cells.

To quantify the degree of the size and deformability overlap, receiver operating characteristic (ROC) curves were plotted and the corresponding area under the curve (AUC) calculated (Fig. S2). Using this approach we determined that enucleated cells are 100% separated (AUC = 1) from the nuclei population in terms of size (Fig. S3b) and deformability (Fig. S3d). Microfluidic systems have been designed to separate particles in the same range of sizes (diameter) as nuclei (6 µm) and enucleated cells (7–10 µm)^24,49^ and the data supports the potential for a size-based separation as a route for nuclei removal. Figure S3a shows that within a population classified in terms of size as enucleated cells, 17% of those cells belong to the nucleated cell population (AUC = 0.83). Therefore size-based separation between nucleated and enucleated cells would be possible but would result in a significant contamination. The deformability difference between enucleated and nucleated cells (Fig. S3c) is close to 100% (AUC = 0.99) suggesting the feasibility of deformability to separate these populations.

### Young’s modulus measurements for enucleated and nucleated cells and nuclei with AFM

AFM is the current gold standard for the biomechanical characterisation of cells. AFM imaging was employed to confirm the size based analysis. Furthermore, AFM force-distance curves were utilised to analyse the differences observed by RT-DC in deformability and convert from the circularity measure of deformability to elastic modulus, which is a useful measure for the design of separation systems.

**Figure 2:**
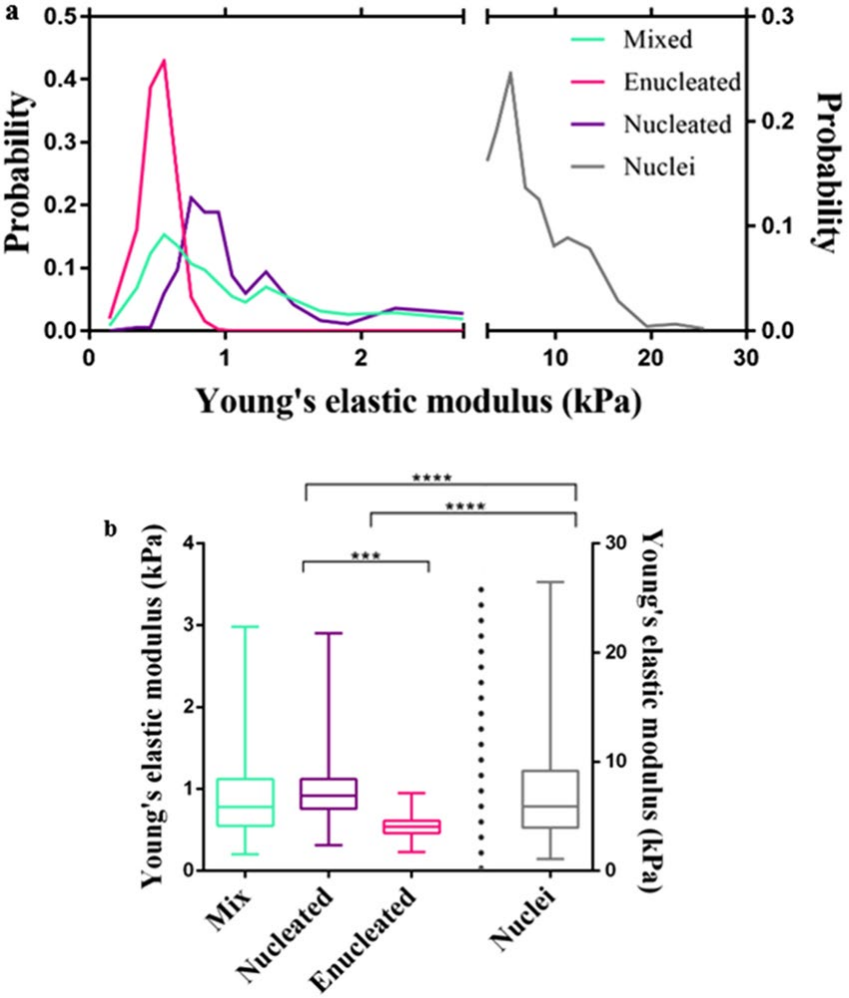
**(a)** QI mode of AFM is applied to measure the elastic properties of umbilical cord CD34+ cells during their differentiation process along with the nucleus extruded from the cells. The Young’s elastic modulus is calculated using the Hertz-Sneddon model for a conical tip. Derived elastic modulus is plotted versus probability for the nucleated, enucleated and a mixture of nucleated and enucleated cells at D14 of cell differentiation along with the nuclei. **(b)** Box and whisker plot summarising the Young’s elastic modulus of the different subpopulations. The line in the box represents median, the box itself 25 and 75% (upper and lower quartile) and whisker extreme values (60 samples for mix and nuclei, 25 samples for nucleated and enucleated cells; ***p < 0.001 ****p < 0.0001), where each box presents the distribution of data from lower and upper quartile with a line in each box shows the median. The whiskers represent the lowest and highest extreme values.

Using AFM Quantitative Imaging (QI) mode and a sharp conical tip, the elastic properties of 60 D14 cells from a mixed culture were measured along with individual populations, isolated by FACS, of 25 nucleated and 25 enucleated cells and 60 nuclei (Fig. 2). As discussed in previous work, AFM elastic measurements are position-dependant^50,51^ which means based on the location indented on the cell, derived elastic modulus can be different from another location on the same cell. These changes in the elastic modulus across the cell are due to the presence/absence of a nucleus and other organelles. Therefore, 64 measurements were taken at different locations of each cell to obtain elasticity changes over the cell, and the derived Young’s elastic modulus was averaged (Fig. 2a). To determine the elastic modulus, calculations were based on the Hertz-Sneddon contact model utilising all derived results from different locations on each cell and all cells in each group (mixed, enucleated and nucleated cells, and nuclei). To study the difference in the elastic modulus of the subpopulations, each range of the elastic modulus was plotted against the probability of each elastic modulus [E] (Fig. 2a). Probability can be defined as the number of occurrences of a targeted elastic modulus divided by the number of occurrences plus the number of failures of occurrences (total possible outcomes) and it is calculated from:

2 $$probability=\frac{number\,of\,events\,within\,targeted\,E\,range}{total\,number\,of\,events\,}$$

The average Young’s elastic modulus for the mixed cells, nucleated and enucleated cells, and nuclei were derived as: 0.91 ± 0.52 kPa (n = 60), 1.04 ± 0.47 kPa, and 0.53 ± 0.12 kPa (both n = 25), and 7.06 ± 4.07 kPa (n = 60) respectively (*cf*. Figure 2b for boxplot summarizing the average and range with the underlying data included in Tables S2 and S3). The elastic modulus of RBCs was reported in the literature as (0.143 ± 0.059 kPa), using spherical indenter^52^. As expected the elasticity of enucleated cells is consequently the closest to the elasticity of RBCs, although still somewhat higher. However, it needs to be mentioned that most cell studies use spherical indenters, which can give a lower elastic modulus when compared directly to a conical tip. It can be noted that biological changes, including a gradual decrease in the number of organelles, continue after the enucleation process resulting in further softening of the cells^53^.

This data is in accordance with the RT-DC data, showing that the enucleated cells are the most deformable whereas the nuclei are stiffest. Furthermore, as also shown by AFM, there is a significant difference between the derived Young’s elastic modulus of nucleated cells and nuclei (p < 0.0001), enucleated cells and nuclei (p < 0.0001, AUC = ) and nucleated and enucleated cells (p < 0.001), (Fig. 2b). Thus, the AFM data confirms the RT-DC finding that deformability is a potential homogeneity marker for downstream sample processing and quantifies the elastic modulus difference by creating ROC curves and calculating the AUC = 0.94 (Fig. S4) for nucleated and enucleated cells. Existing separation techniques based upon deformability enables the sorting of cells using differences in their Young’s elastic modulus. For instance it has been demonstrated that for a mixed cell culture media, a mixture of four cells, MDA MB 231, HL60, MCF 7 and HeLa, with Young’s elastic modulus of 1 kPa, 2.7kPa, 3.4 kPa and 13.5 kPa could be sorted using the stiffness-based sorting microfluidic channel with an efficiency ranging from 70 to 85%^54^.

AFM images from individual populations of nucleated cells, enucleated cells and nuclei were utilised to study the morphological differences among subpopulations. As also demonstrated by the RT-DC data, nuclei were observed to be smaller both in height and width in comparison to the other two subpopulations (Fig. S5a). Comparing enucleated and nucleated cells (Fig. S5b and c) the nucleated cells are slightly higher and narrower than the enucleated cells since on the poly-D-lysine coated petri dish the enucleated cells spread out more.

#### Correlation between mechanotype and biology

**Figure 3:**
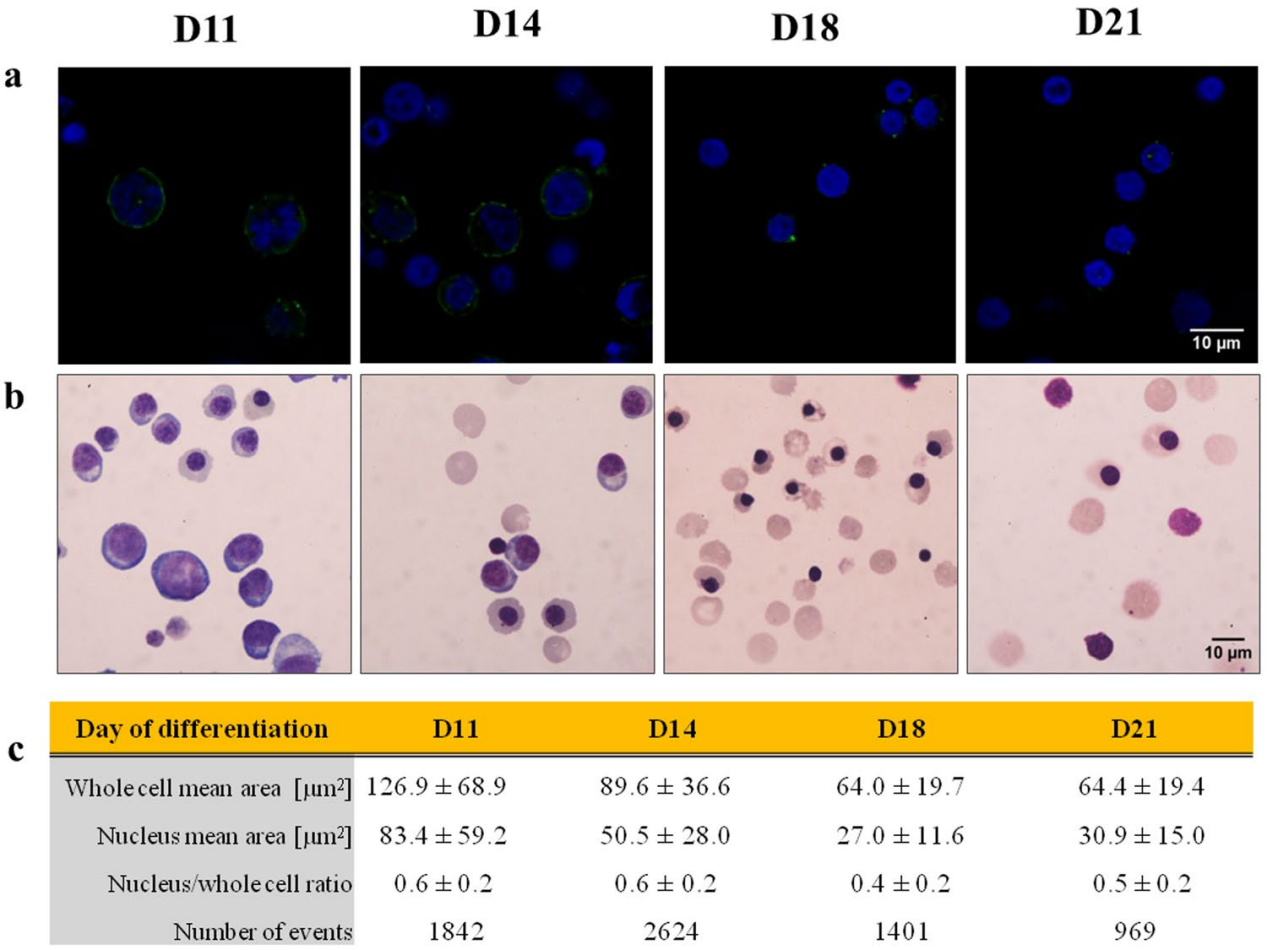
To visualise changes in cell morphology over the course of the differentiation procedure (Day 11, 14, 18 and 21) **(a)** cytoskeletal protein F-actin (green) and nuclei (blue) were stained with fluorophores and **(b)** cytoplasm and nuclei were stained with Romanowsky stain. Scale bars represent 10 µm; **(c)** cytospin images of Rmanowsky’s stained cells were analysed using Matlab for cell and nucleus area (mean ± SD) and nucleus/ whole cell ratio were calculated (mean ± SD).

Cell deformability is determined by the structural components of the cell, particularly the cytoskeleton^55^. The cytoskeleton is a protein network (mostly actin and spectrin in RBCs^56^) supporting the cell structure and providing mechanical integrity to the cells. This network underlies the cell membrane and connects with the nucleus by extending through the cytoplasm and plays a crucial role in the enucleation, which is a dynamic process lasting approximately 10 min^57^. During the process, F-actin and myosin filaments undergo re-arrangements to facilitate the act of enucleation. Paraformaldehyde fixed cell samples were stained at D11, D14, D18 and D21 for F-actin (Fig. 3a), one of the most abundant cytoskeletal protein, and imaged. At D11 and D14 F-actin assembles into a uniform shell surrounding the cell interior. By the end of the differentiation protocol, at D18 and D21, the shell becomes thinner and less visible with small aggregates visible, which support the observed softening of nucleated cells between those days. Observed thinning is in agreement with literature evidence, where cytoskeletal protein levels were quantified by Western Blot^36^. It was reported that actin is downregulated during erythropoiesis with α-spectrin and β-spectrin expressed in higher quantities. Together, those rearrangements are believed to be an adaptation of RBCs to change their shape under the applied shear stress they experience during the vascular circulation, without haemolysis. When the lack of a nucleus is the main feature providing optimal cell deformability for oxygen transfer in tissues^58^. The presence of a nucleus, its changing characteristics and eventual expulsion throughout differentiation could contribute substantially to the overall cell mechanical behaviour^59^.

**Figure 4:**
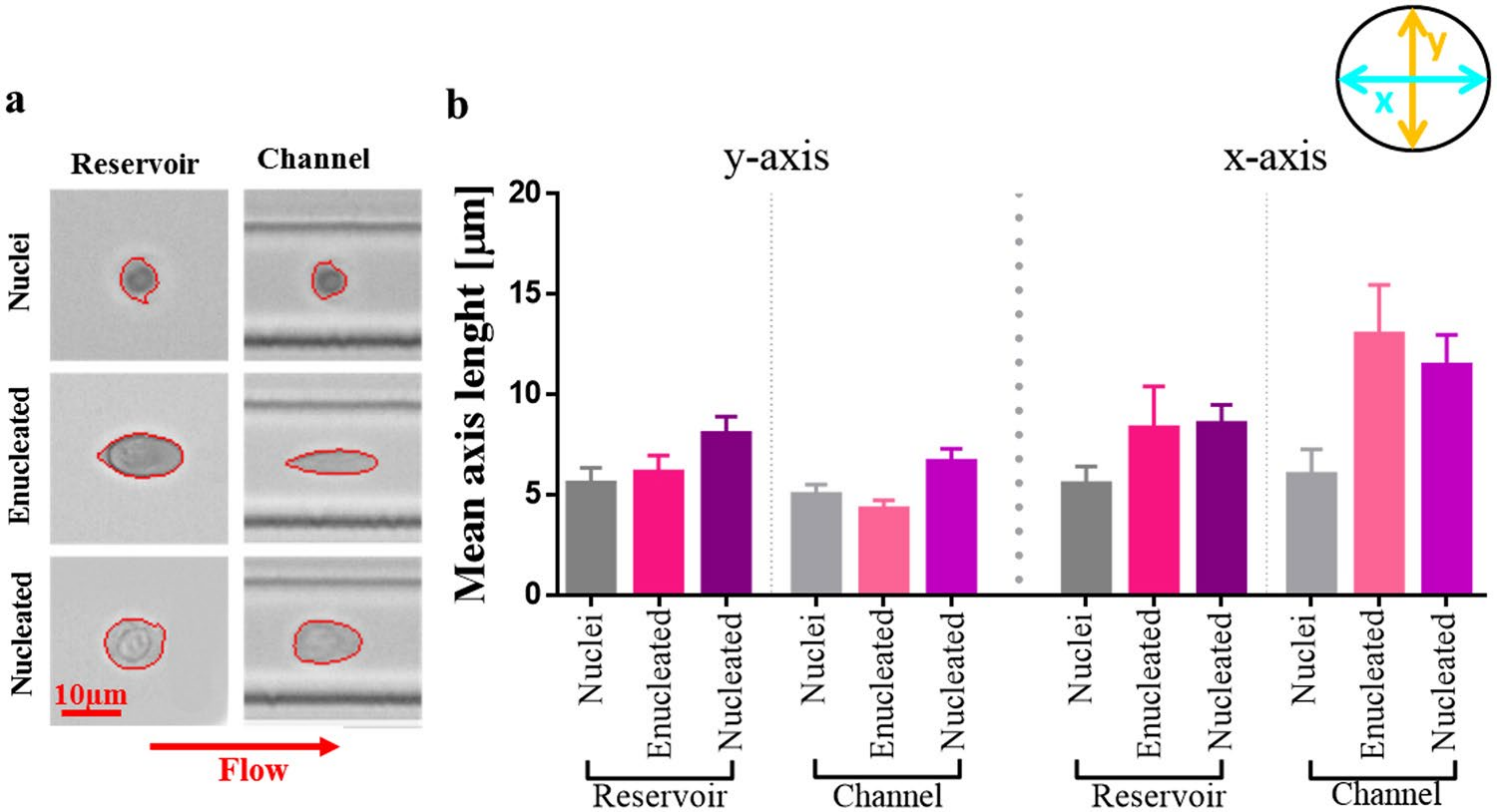
Cells at D14 were sorted on FACS into enucleated, nucleated cells and nuclei populations. Their minor and major axis (y and x respectively) were assessed by RT-DC image analysis. **(a)** Measurements were captured in both the reservoir (with negligible degree of shear stress) and in the channel (20 µm width) where shear stress exerted on cells causes them to deform from their original shape. **(b)** Changing x- and y-axis length for pure populations, in the reservoir and in the channel, were captured. Bar graph represents mean axis length with error bars showing SD from the mean.

The contribution of the nucleus to the mechanotype is determined by its relative size^60^. To assess the nucleus/whole cell ratio, cytospin cell slides prepared at D11, D14, D18 and D21 were stained with rapid Romanowsky stain and visualised using a bright field microscope (Fig. 3b). Approximately 1000 events per condition were analysed and results are presented in Fig. 3c. Nuclei become smaller over time and at D11, on average, they constitute around 60% of the cell. The cytoplasm becomes simplified at later days and the nucleus is more compacted and smaller (50% for D14 and 40% for D18). We further explored the contribution of nuclei to the mechanical behaviour of cells by looking at cell elongation within the RT-DC microfluidic device under shear stress (Fig. 4a and b). In the RT-DC system, cells first pass through a reservoir section to then be forced through a smaller channel where a sheath flow induces a shear stress on the cells (Fig. S6). As presented in Fig. 4, the y-axis for enucleated and nucleated cells is larger than nuclei in the reservoir. When cells enter the main channel, the nuclei y-axis remains unchanged while the enucleated y-axis is shortened and the nucleated y-axis remains above the length of the nucleus. The presence of the nucleus seems therefore to constitute a barrier to the capability of a cell to deform under shear stress. To verify if it would be possible to separate enucleated and nucleated cells based on the degree of deformation they undergo under hydrodynamic stress, the length of the y-axis of enucleated and nucleated was compared. The measurement was obtained in the reservoir section of the device where cells experience shear stress and by creating a ROC curve and calculating the AUC = 1 (Fig. S7) we see that there is no overlap in the y-axis length. Deformability and size are coupled and the cell size revealed by their original shape change under applied stress could constitute a basis for cell separation (e.g. by passing through a filter with a cut-off size smaller than the length of y-axis for nucleated cells).

#### Conclusion and Outlook

Regenerative medicine, and cell therapy, in particular, are seen as a potential route to revolutionize medicine and to improve healthcare for patients who currently have either limited or no treatment options. The *in vitro* produced RBCs are seen to address needs in transfusion medicine.

For the wide-spread adoption of mRBCs, and indeed other cell therapies, challenges related to cell source, maturation and viability need to be addressed by biologists, while advancements in cell processing technologies will be required to manufacture those cells in meaningful quantities and achieve satisfactory purity. Label-free separation techniques based on mechanical phenotype differences offer a promising route to large-scale purification. However, to design optimal downstream processing protocols we first need to understand the mechanical characteristics of subpopulations within the samples, how these relate to molecular and architectural changes and how those changes evolve with cell-state and progressing differentiation protocols. Thus, here we characterised the mechanical properties (size and deformability) of CD34+ cells from cord blood as they undergo *in vitro* erythropoiesis. Cell size and deformability are highly dynamic features, significantly changing during the differentiation process. Based on our observations, using both novel high-throughput techniques (RT-DC) and AFM, cells become smaller and softer between D11 and D18 as different cell subpopulations emerge. Our staining results, linked with data from the literature, suggest that this phenomenon is driven by changes in the nucleus properties, which is expelled towards the end of the protocol, coupled to morphological changes in the cytoplasm and cytoskeleton.

Several key findings emerged from our mechanotype analysis. Firstly, there is no overlap between the sizes of the nuclei and the cells (both enucleated and nucleated). Data confirmed a 100% separation for both size and deformability. It could, therefore, be possible to design label-free systems to remove the nuclei exploiting the 2 µm difference in the average diameter of the cells and the nuclei at D18, a size at which there is plenty of evidence in the literature for successful separation approaches targeting different applications^49^.

Secondly, since the data showed a significant overlap of the enucleated/nucleated cell populations in terms of size, the cell mechanotype marker of size might thus not be practical. Thirdly, we demonstrated a significant difference in deformability (from ROC curves AUC = 99%) and more specifically in Young’s elastic modulus (0.51 kPa difference on average at the end of the protocol) between the enucleated/nucleated cell populations. In order to purify enucleated cell from nucleated cells, methods which exploit either, or both of, the observed differences in cells Young’s elastic modulus and deformation under shear stress could be utilised.

Overall, our data provide the first quantitative information regarding the mechanotype of CD34+ cells undergoing differentiation into manufactured RBCs, which could assist in the design of robust label-free purification approaches.

## Materials and Methods

### Sample Preparation

#### Cord CD34+ Stem Cell Culture

All methods were carried out in accordance with relevant guidelines and regulations and were approved by the Heriot-Watt Engineering and Physical Sciences Ethics Committee as well as the Heriot-Watt Engineering and Physical Sciences Biosafety Review. The cells used in this work were commercially obtained from donated cord blood and therefore consented to the research use. The CD34+ hematopoietic stem cells were primarily purchased from Stem Cell Technologies, expanded and cryopreserved after 6 days in culture using a variation of the method previously described^61^. A master cell bank from one healthy donor was created with D6 cells cryopreserved in fresh medium supplemented with 30% Knockout Serum Replacement and 10% DMSO. Cells at day 6 were resuscitated, washed and re-cultured in fresh pre-warmed growth medium: Iscove’s basal medium (VWR, cat. BCHRFG0465), 5% human AB+ Serum (Sigma Aldrich, cat. H4522), 3 U/ml heparin (Sigma Aldrich, cat. H5515), 10 µg/ml insulin (Sigma Aldrich, cat. 19278) and 200 µg/ml human holotransferrin (VWR, cat. 616397-500) supplemented with 60 ng/ml recombinant human stem cell factor (SCF) (PeproTech, cat. 300-07), 5 ng/ml recombinant human IL-3 (PeproTech, cat. 200-03), 3 U/ml erythropoietin (EPO) (clinical grade material, Roche) and 1 µM hydrocortisone (Sigma Aldrich, cat. H0888) until day 8. At day 8 cell culture medium was replaced with fresh ISHIT cell culture medium supplemented with 10 ng/ml SCF, 3U/ml Erythropoietin, 1 µM Hydrocortisone and 300 µg/ml Transferrin and cultured until day 14. At day 14, a cell count was performed using the trypan blue exclusion technique, cells centrifuged at 300 g for 5 min and re-seeded in fresh culture medium containing 3 U/ml erythropoietin and 300 µg/ml holotransferrin. After a further four days of culture (day 18) cells were re-seeded in fresh medium (the same composition as day 14). Each time cells were harvested, they were transferred into a centrifuge tube (Corning, UK) and centrifuged at 300 g for 5 min. All cell culture manipulations were carried under aseptic conditions in a cabinet with a laminar air flow.

### Flow Cytometry

Cells were assayed for expression of CD235a (Glycophorin A) and for the presence of a nucleus using flow cytometry during differentiation. At each control time point: day 11, 14, 18 and 21 (D11, D14, D18 and D21), cells were collected and counted, centrifuged at 300 g for 5 min and re-suspended in basal medium supplemented with 0.5% BSA at a concentration of 1 × 10^7^ cells/ml. To each 100 µl aliquot of cells, 0.625 µl of FITC-conjugated Mouse Anti-Human CD235a (BD, cat. 559943) and 0.5 µl of 5 mM DRAQ5™ Fluorescent Probe (BD, cat. 564902) was added to obtain a final concentration of 5 µM. Cells were incubated for at least 30 min at room temperature in darkness. The excess fluorescent stain was not removed to prevent cell damage. Cells were analysed on a BD FACSCalibur within two hours of staining and raw data analysed using FlowJo V10 CL. The same staining strategy was used for cell sorting by FACS (FACSAria IIu flow cytometer, Beckton Dickinson Immunocytometry Systems (BD, UK) running BD FACSDiva v6 Software.

### Mechanotype Characterisation

#### Real-Time Deformability Cytometry

Cells size and deformability changes were assessed using a Real-Time Deformability Cytometer (RT-DC). Measurements were performed as described in Nat. Methods 2015^43^. In our work, a PDMS chip with a 20 µm × 20 µm cross-section channel was used. Prior to measurements, cells were harvested by centrifugation at 300 g for 5 min and resuspended in a 0.05% methylcellulose solution at 1–2 × 10^6^ cells/ml. Cells were pumped into the channel at 0.12 µl/min. RT-DC data were analysed using original RT-DC software ShapeOut 0.6.9 (available at www.zellmechanik.com). Using the polygon tool built in the software, area and deformability values were obtained separately for day 11, 14, 18 and 21 as well as for separate subpopulations (enucleated cells, nucleated cells and nuclei). Data were then extracted from the software and further analysed using MatLab R2016b and GraphPad Prism 7. The significance of the results was calculated using a standardized mixed model that assumes non-normal distribution with three replicas of each experiment (Fig. S8). The receiver operating characteristic curves were generated and the area under the curve was calculated using a customised MatLab script (MatLab R2016b).

#### Atomic Force Microscopy (AFM) -Cell preparation

To prepare cells for AFM measurements, a concentration of 2 × 10^6^ cells/ml was seeded onto poly-D-lysine hydrobromide (Sigma Aldrich cat. P6407, UK) coated AFM petri dishes. The dishes were then incubated for 1 minute to allow for adequate cellular attachment. Cell seeded sample dishes were then gently rinsed with 1 ml of Hank’s Balanced Salt Solution (HBSS, pH = 7) (Merk Millipore cat. H9394, UK) to remove any loosely and/or unattached cells. 2 ml of HBSS was then added to the sample dish, which was then mounted onto the BioCell™ stage, which maintains the cell sample and HBSS media at 37 °C for the duration of the AFM experiment^52^. The same protocol was applied for nucleus preparation.

### AFM quantitative imaging mode

AFM measurements were carried out using the NanoWizard III Bio AFM (JPK Systems, Berlin, Germany), mounted on a Zeiss Observer D1 inverted optical microscope placed on top of a Halcyonics i4 anti-vibration table. For all experiments, silicon coated HQ: NSC36/Cr-Al series conical probe cantilevers, (MicroMasch, UK) half cone angle of 20° and tip radius of ~8 nm with spring constant of 0.01 N/m were used. In AFM measurements, obtained elastic modulus are highly dependent on the shape of the tip, indentation depth, cell preparation protocol and applied contact mechanical model. For instance, if comparing indenting a cell with a conical or a spherical shape indenter, the derived elastic modulus from the conical tip is higher than the one obtained from the spherical tip. This can be due to the fact that the conical tip provides more local elastic properties while the spherical tip presents the overall elastic properties^62^. The thermal noise method is used to determine the spring constant of the cantilever. Quantitative Imaging (QI) mode with the map size of 8 × 8 indexes is used to record force-distance curves from the whole area of each cell. For all experiments, the indentation speed was kept at 5 μm/sec, with the applied force in the range of 0.8–1 nN, depending on the differentiation date.

#### Elasticity analysis for AFM

Obtained force-distance curves were analysed for the elasticity measurements using the JPK data processing software version spm-5.0.69. Based on the map size of 8 × 8 indexes, 64 force-indentation curves per cell were recorded. The curves, which were from the AFM dish coating and/or residues within the culture media were discarded from the analysis. Due to the shape of the chosen cantilever probes, the Hertz-Sneddon contact model (for the conical probe geometry) was applied to determine the elastic modulus of the cells for each force-displacement curve obtained^63,64^.

3 $$F(\delta )=\frac{2\,E\,tan\alpha }{\pi (1\,-\,{\nu }^{2})}{\delta }^{2}$$

Where F is an applied force, E is Young’s elastic modulus, α is a half cone angle of the AFM tip, δ is the indentation depth and ν is the Poisson ratio.

For use of the Hertz-Sneddon contact model to derive the Young’s elastic modulus of the cells, cells were assumed to be homogeneous, isotropic and semi-infinite bodies. Since the approach curves were not affected by the adhesion force, only the approach curves were used for the elasticity analysis. For each force curve, the baseline correction was carried out by calculating the average value of the baseline and subtracting it from the whole curve. The Hertz-Sneddon contact model was fitted approximately from the point where the force curve starts increasing.

### Biological Characterisation

#### Cell morphology- Cytospin

To visualise cells morphology and structure, cells were transferred onto microscope slides using a cytocentrifuge then fixed and stained using Giemsa-Wright staining (Rapid Romanowsky Stain Pack, TCS Bioscience, cat. SW167/500). Cells at selected time points (D11, D14, D18 and D21) were harvested by centrifugation at 300 g for 5 min and resuspended at 2 × 10^6^ cells/ml in Dulbecco’s PBS−/−. 100 µl of cell suspension was transferred into a cytocentrifuge cell funnel and centrifuged at 450 rpm for 4 min in a cytocentrifuge (Cellspin I Tharmac, Germany) to transfer the cells onto the slide. Slides were then air-dried for 15 min, fixed and stained according to the manufacturer’s instructions. After staining, slides were air-dried then fixed with DePeX mounting medium (Sigma Aldrich, cat. 06522**)**. Slides were photographed for further image analysis using an EOS 60D Canon camera (Canon, UK) mounted on an AXIO Scope.A1 Zeiss microscope (Zeiss, Germany) at ×100 magnification. Images were analysed in Matlab R2016b using a custom-made script.

### Widefield Microscopy

To study the cytoskeletal changes in CD34+ cells undergoing *in vitro* erythropoiesis, cells at day 11, 14, 18 and 21 were fixed in 4% paraformaldehyde (PFA) (ThermoFisher Scientific, cat. 28906) and imaged using a widefield microscope Olympus IX-81 TIRF+ with a ×150 1.4 NA immersion oil lens and EMCCD camera (Hamamatsu, UK). First cells were stained for 30 min at room temperature (RT) against F-actin with Actin Creen 488 Ready Probes reagents (Life Technologies, cat. R37110) then cells were transferred onto poly-D-lysine hydrobromide (Sigma Aldrich, cat. P6407, UK) coated cover slips to attach. Two minutes before the end of the incubation time, Hoechst 33342 (Sigma Aldrich, cat. 14533) was added to a final 10ng/ml concentration. After 2 minutes the cover slip was gently washed with PBS−/− to unattached free-floating cells. Twenty stack images per differentiation day (D11, D14, D18 and D21) were collected. Obtained stack images were deconvoluted with the Huygens Professional software.

### Data availability

The datasets generated during and/or analysed during the current study are available from the corresponding author on reasonable request

## Electronic supplementary material

Supplementary Information
