# Supplementary material for: High-throughput assessment of mechanical properties of stem cell derived red blood cells, toward cellular downstream processing
Source: Sci Rep. 2017 Oct 31;7:14457. doi: 10.1038/s41598-017-14958-w (PMC5663858; doi:10.1038/s41598-017-14958-w)
Supplement: Supplementary file 1 — Supplementary Information [file 41598_2017_14958_MOESM1_ESM.pdf]

High-throughput assessment of mechanical properties of stem cell derived red blood cells, toward cellular downstream processing

Ewa Guzniczak[1*] & Maryam Mohammad Zadeh[1], Fiona Dempsey[2], Melanie Jimenez[3], Henry Bock[1], Graeme Whyte[1], Nicholas Willoughby[1], Helen Bridle[1]

[1]Heriot-Watt University, School of Engineering and Physical Science, Department of Biological Chemistry, Biophysics and Bioengineering Edinburgh Campus, Edinburgh EH14 4AS

[2] MedAnnex Ltd, 1 Summerhall Place, Techcube 3.5, Edinburgh, EH9 1PL

[3] University of Glasgow, School of Engineering, Biomedical Engineering Division, Glasgow G12 8QQ

*eg100@hw.ac.uk

Supplementary Information

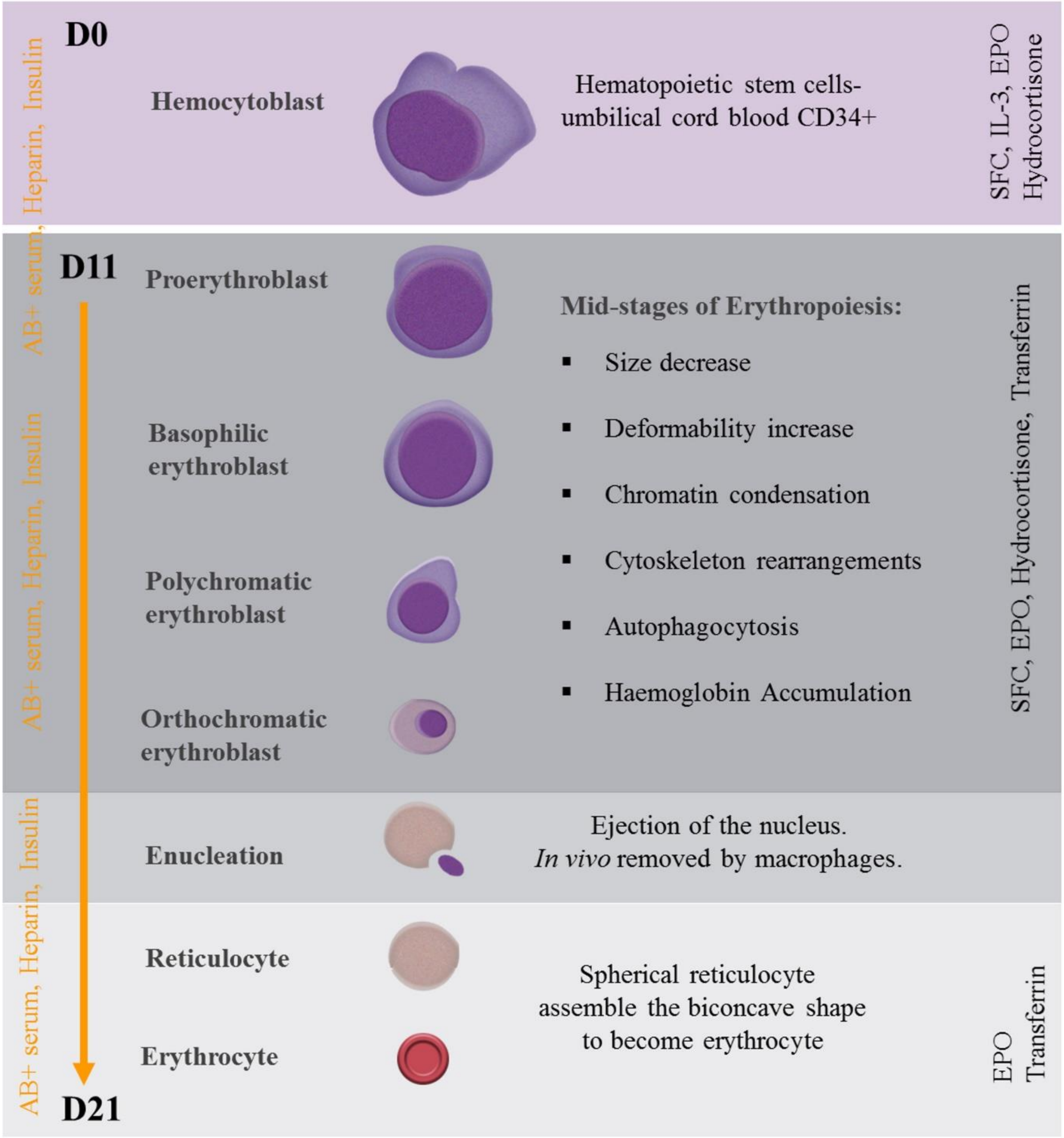

Fig. S1 Schematic showing stem cells differentiation into red blood cells. The process starts (day 0) with hematopoietic stem cells, CD34+ subpopulation obtained from umbilical cord blood. Basal medium always contains 5% AB+ human serum, Heparin and Insulin. Over the course of the differentiation protocol cell culture medium is supplemented with different cytokines and growth factors. Those changes in medium composition push stem cell through distinct developmental stages (*in vitro* recapitulation of *in vivo* erythropoiesis). By day 18 cells lose their nuclei to become reticulocytes, which maturate into erythrocytes by day 21.

Methodology			Throughput	Ref.
Atomic Force Microscopy (AFM)	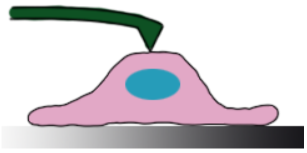	Using scanning probe (cantilever with a sharp tip) for poking/ touching surface of the sample with a resolution of fractions of nanometres for viscoelasticity measurement	< 100 cells/ hr	[1,2,3,4,5,6,7,8]
Optical stretcher (tweezers and traps)	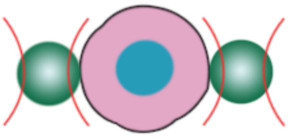	Optical stretcher is a dual-beam optical trap where a particle/ cell is trapped by optical forces generated by two opposite laser beams.	< 100 cells/ hr	[9,10]
Magnetic twisting cytometry/ tweezers	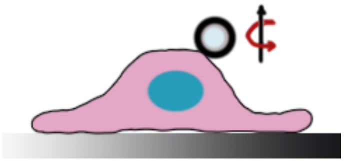	Magnetic bead is strongly attached to cell surface and by applying and manipulating magnetic field a portion of cell is twisted.	< 100 cells/ hr	[11,12,13]
Micropipette aspiration	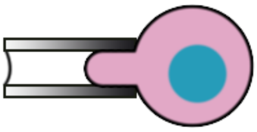	Applying suction pressure on a cell membrane and measuring it extension into the bore of glass micropipette. Measures viscoelasticity	< 100 cells/ hr	[14,15,16]
Hydrodynamic cytometry	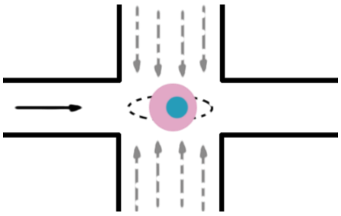	Deforming cell at high strains in a stretching extensional flow filed within microfluidic device	Thousands cells/ min	[17]
Real-time deformability cytometry	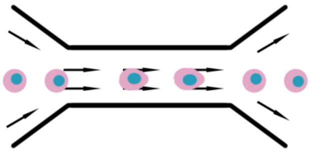	Cells are deformed by pressure gradient and shear stress in a constriction in a contact-less manner	Thousands cells/ min	[18,19,20]
Transit through constrictions	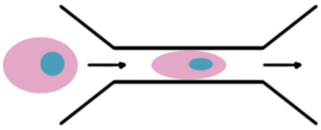	It a microfluidic device cells are pushed through custom-made constrictions smaller than cell size , it can be aspiration-, fluid- and electrically induced process	1-100 cells/ s	[21,22,23]

Table S1. Summary of commonly used currently available methodologies for cell meachanotyping. The table contain the name of a technique, a graphical representation of the method accompanied by a description, throughput (a number of cell that could be assessed in a given time) as well as a selection of references, where a given method was used for assessing cell mechanical properties.

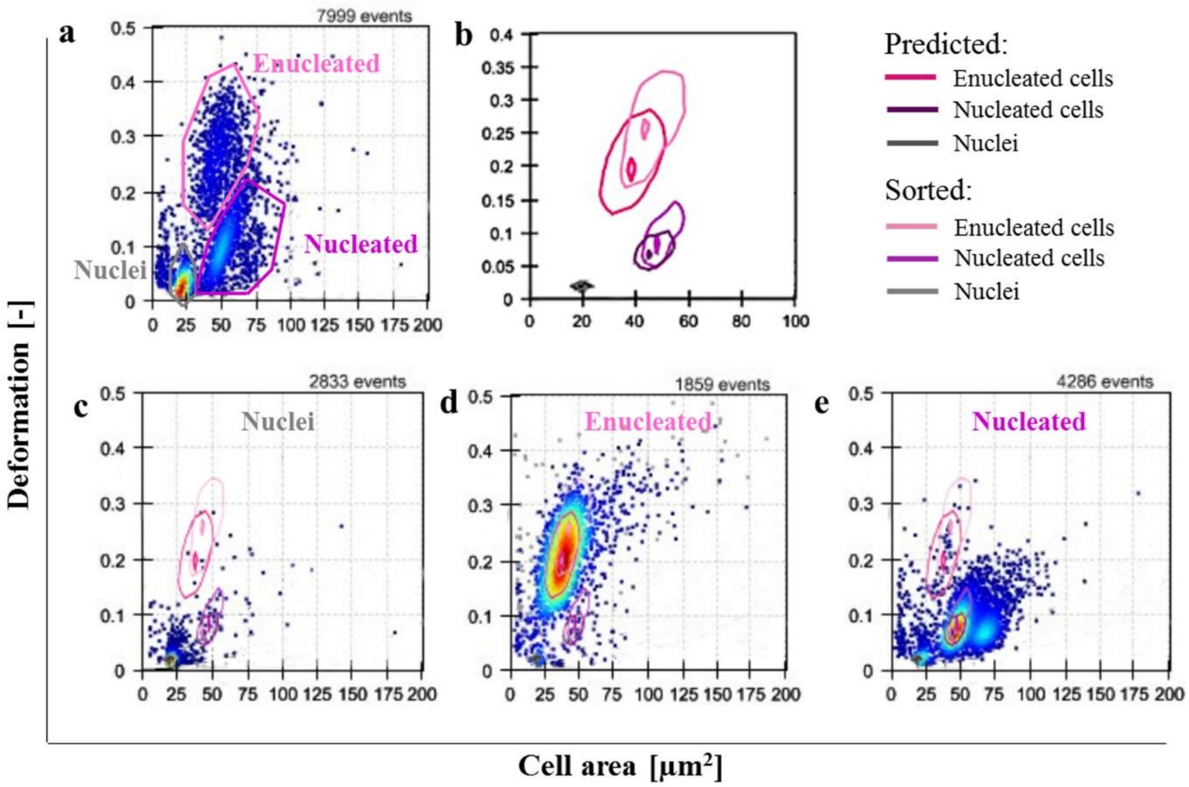

Fig. S2 Gating strategy. Presented data was generated using RT-DC with a 20 µm × 20 µm cross-section channel and a flowrate of 0.12 µl/ min. Each dot represents the measurement for one cell at D14, with the total number of events indicated on the top of each diagram. Grey isoelasticity lines on the scatter plots represent a predicted cell deformability for cells of the same elasticity and different size [43].On the first dot plot **(a)** three subpopulations can easily be distinguished, corresponding to enucleated and nucleated cells as well as nuclei. Properties for nuclei **(c),** enucleated cells **(d)** nucleated cells **(e)** were assessed separately on the RT-DC after being sorted into pure subpopulations by FACS. Predicted populations from **(a)** were gated using the polygon tool in the ShapeOut software and their contour were compared with the contour plots **(b)** generated for the pure subpopulations **(c, d, e).** The contour for each population consist of a smaller inner contour representing 50% density and a larger outer contour corresponding to 90% density.

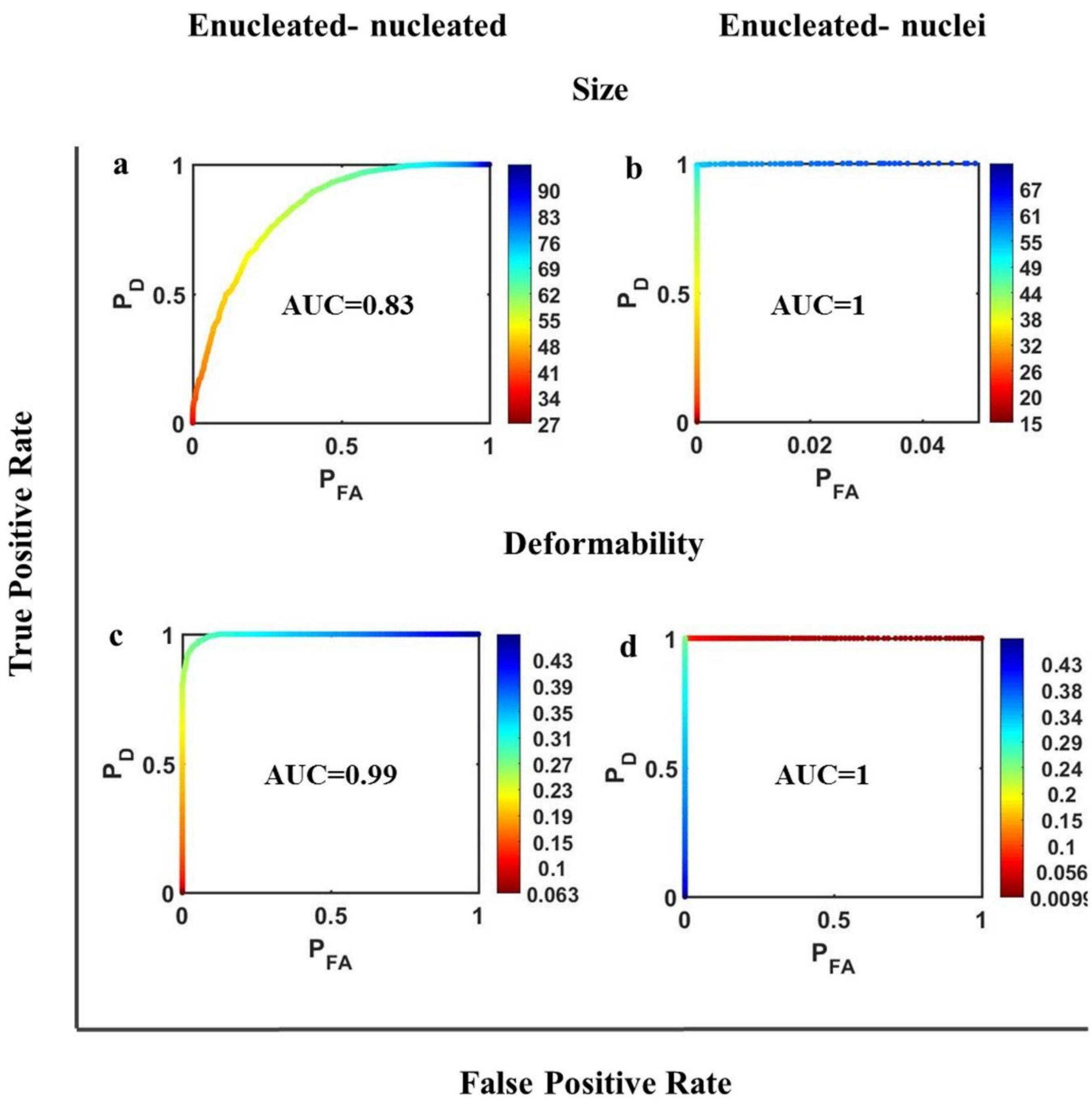

Fig. S3 Receiver Operating Characteristic (ROC) curves were plotted for size (expressed as area [µm2]) and deformability [-] for enucleated versus nucleated cells (left) and nuclei (right) at D18.The True Positive Rate is defined as the number of enucleated cells measured for a certain cut-off point (size or deformability) and divided by the total number of enucleated cells. The False Positive Rate is the corresponding number of nucleated cells (resp. nuclei) divided by the total number of enucleated cells (resp. nuclei) for the same cut-off. The Area Under the Curve (AUC) was calculated to quantify the size overlap between **(a)** enucleated and nucleated cells population, **(b)** enucleated cells and nuclei as well as deformation overlap between **(c)** enucleated and nucleated cells and **(d)** enucleated cells and nuclei populations. Curves were color-coded to correspond to cell area expressed in µm2 for (a) & (b) and cell deformability (c) & (d), which is a dimensionless value.

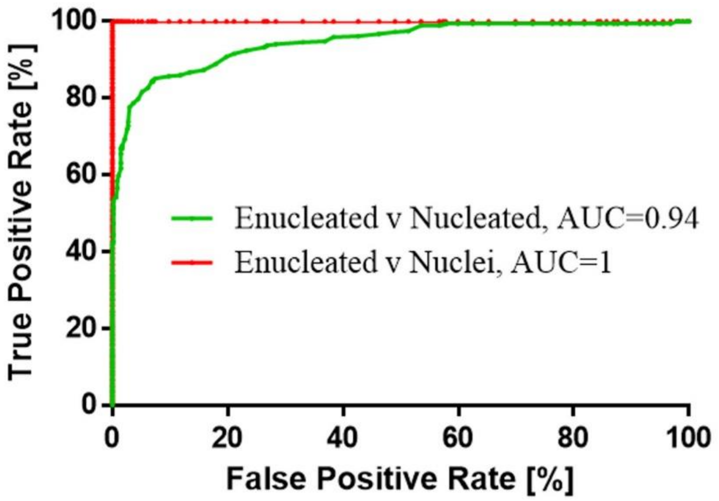

Fig. S4 Receiver Operating Characteristic (ROC) curves were plotted Young modulus measured for enucleated and nucleated cells as well as nuclei. The True Positive Rate is defined as a number of enucleated cells measured for a certain elasticity cut-off point and divided by the total number of enucleated cells. The False Positive Rate is the corresponding number of nucleated cells (nuclei respectively) divided by the total number of enucleated cells for the same cut-off. The Area Under the Curve (AUC) was calculated to quantify the elasticity difference between enucleated and nucleated (green) and enucleated cells and nuclei (red).

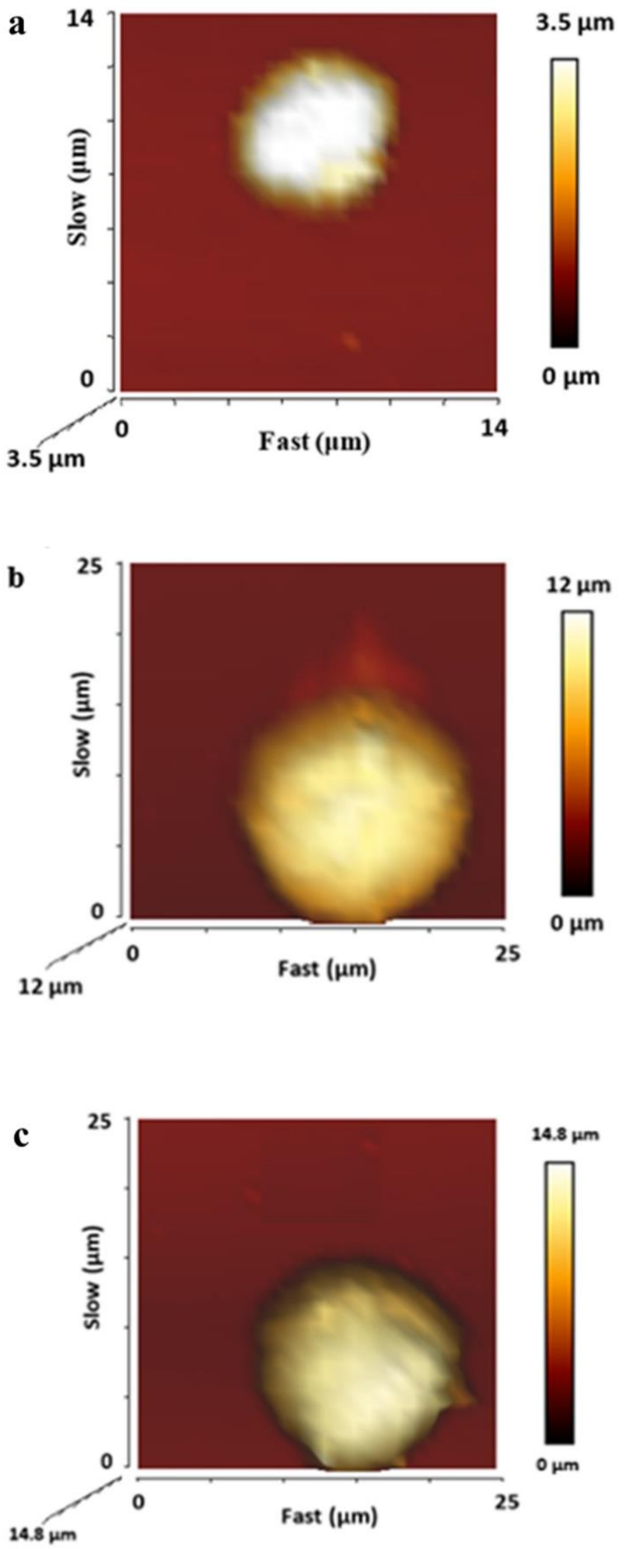

Fig. S5 AFM images of the D14 cells and nucleus are provided by using the QI mode of AFM with the map size of 32×32 indexes **(a)** nucleus **(b)** enucleated cell and **(c)** nucleated cell. The height channel was selected for image demonstration to visualise the changes in the size of the cells due to the enucleation process. The bars on the right side of the images shows the height range of the cells and nucleus following deformation from the probe.

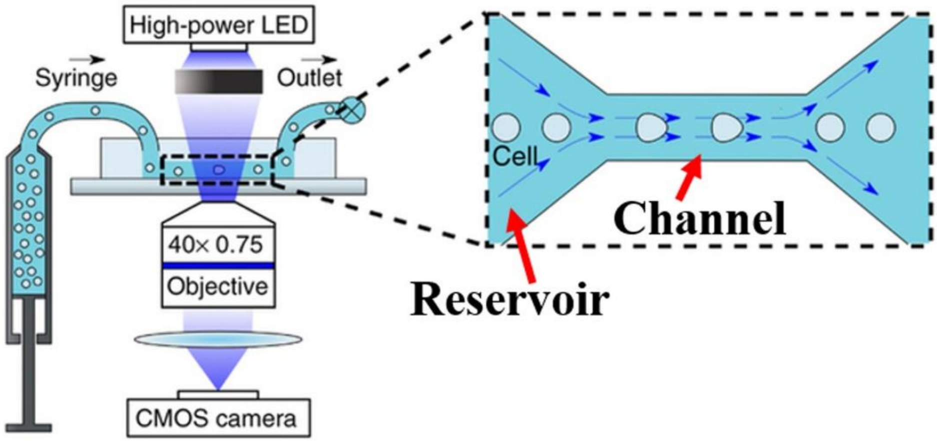

Fig. S6 A schematic adapted from Otto, Oliver, et al. "Real-time deformability cytometry: on-the-fly cell mechanical phenotyping." N*ature methods* 12.3 (2015): 199-202. RT-DC consist of syringe pump that pumps cell suspension into a PDMS chip mounted on a microscope. Cells enter the chip through the reservoir section, next they are concentrated into a single stream by sheath fluid and enter the measurement channel where they experience deformation due to applied shear stress and pressure. The degree of deformation is measured based on high-throughput real-time image analysis.

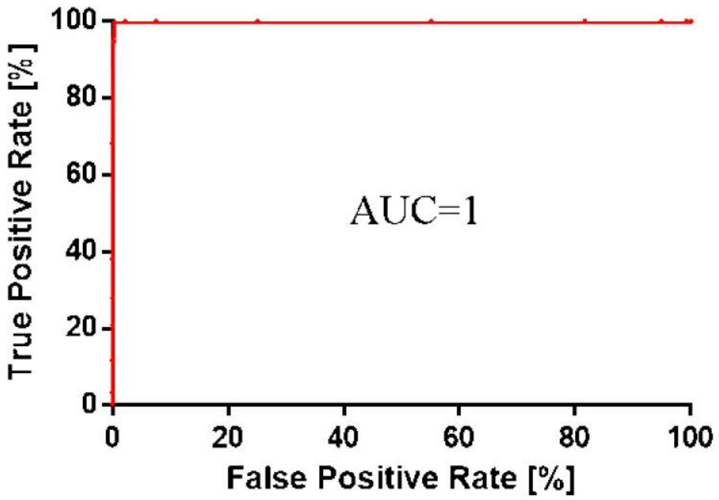

Fig. S7 Receiver Operating Characteristic (ROC) curves were plotted to compare enucleated and nucleated cells y-axis overlap (Fig. 4). The True Positive Rate is defined as length of y-axis for number of enucleated cells measured for a certain elasticity cut-off point and divided by the total number of enucleated cells. The False Positive Rate is the y-axis of corresponding number of nucleated cells divided by the total number of enucleated cells for the same cut-off. The Area Under the Curve (AUC) was calculated to quantify the overlap between the measured length of the y-axis for enucleated and nucleated cells.

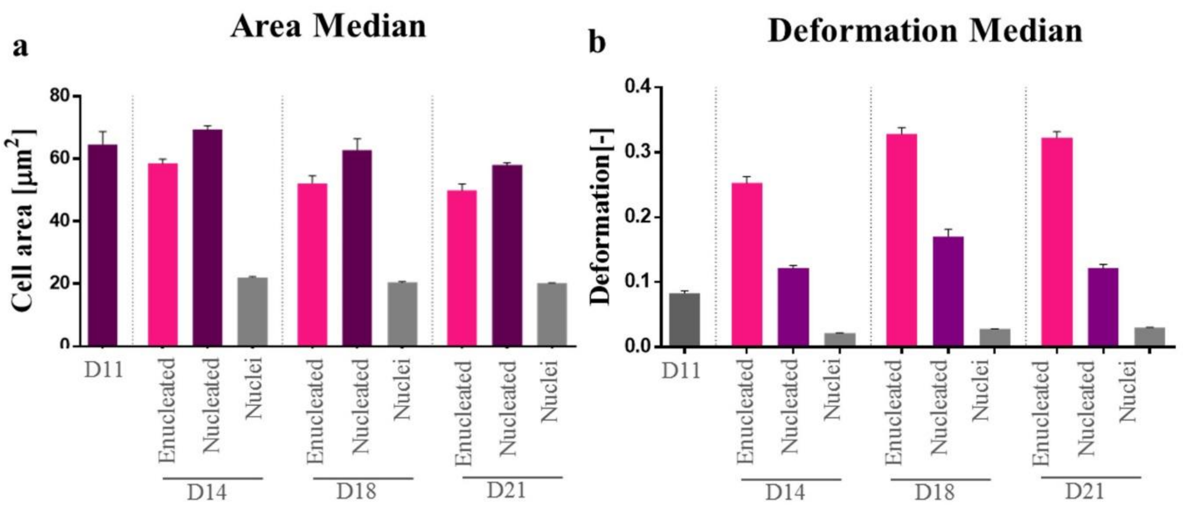

c

Area		D11	D14 enucleated	D14 nucleated	D14 nuclei	D18 enucleated	D18 nucleated	D18 nuclei	D21 enucleated	D21 nucleated	D21 nuclei
Median	Mean	64.63	58.57	69.43	22.10	52.23	62.87	20.43	49.90	58.00	20.27
	Std. Deviation	4.12	1.36	1.16	0.17	2.33	3.56	0.29	2.07	0.78	0.06
25% Percentile	Mean	54.43	52.48	61.07	20.97	47.27	56.17	19.60	45.24	48.97	19.33
	Std. Deviation	2.50	1.43	0.49	0.25	2.28	3.68	0.17	2.04	1.91	0.06
75% Percentile	Mean	83.60	64.55	80.07	23.67	57.27	70.10	21.67	54.73	66.40	22.20
	Std. Deviation	3.68	0.83	0.97	0.15	1.94	3.45	0.42	2.01	1.21	0.20

d

Deformation		D11	D14 enuclated	D14 nucleated	D14 nuclei	D18 enuclated	D18 nucleated	D18 nuclei	D21 enuclated	D21 nucleated	D21 nuclei
Median	Mean	0.08	0.25	0.12	0.02	0.33	0.17	0.03	0.32	0.12	0.03
	Std. Deviation (%)	0.33	1.00	0.40	0.05	1.02	1.15	0.04	0.93	0.59	0.03
25% Percentile	Mean	0.06	0.22	0.09	0.02	0.29	0.13	0.02	0.28	0.08	0.02
	Std. Deviation (%)	0.34	0.60	0.28	0.03	1.21	0.97	0.01	0.91	0.49	0.03
75% Percentile	Mean	0.11	0.29	0.16	0.03	0.36	0.20	0.04	0.36	0.16	0.04
	Std. Deviation (%)	0.35	1.10	0.56	0.14	0.85	1.33	0.10	0.87	0.67	0.03

Fig. S8 Presented bar graphs show mean of area **(a)** and deformations **(b)** medians for three replicas of the RT-DC measurements for cells at D11.and enucleated, nucleated and nuclei populations from D14, D18 D21. Populations were identified from mixed samples by the gating scheme as described in the main body of the text. Error bars represent standard deviation from the mean. All measurements were performed in three replicas for cells at the same days of differentiation protocol. Cells come from three separate batches, from the same master bank created for one donor. Tables **(c)** and **(d)** show averaged medians for cell are and deformability values obtained at three separate occasion. Because deformation values are small, the SD is represented as % for the easiness of reading.

	**Enucleated**		**Nucleated**		**Mix**	
Elastic modulus range (kPa)	Average elastic modulus (kPa)	S.D.	Average elastic modulus (kPa)	S.D.	Average elastic modulus (kPa)	S.D.
**0-0.29**	0.254	0.0213	0	0	0.244	0.02814
**0.3-0.4**	0.355	0.0284	0.31	0	0.358	0.0324
**0.41-0.5**	0.46	0.0296	0.503	0.0028	0.454	0.0291
**0.51-0.6**	0.557	0.0282	0.558	0.0297	0.55	0.0276
**0.61-0.7**	0.65	0.0284	0.65	0.0265	0.65	0.0288
**0.71-0.8**	0.744	0.0293	0.759	0.0294	0.755	0.0308
**0.81-0.9**	0.853	0.0304	0.853	0.0294	0.857	0.0272
**0.91-1**	0	0	0.952	0.0296	0.956	0.0271
**1.01-1.1**	0	0	1.062	0.0313	1.053	0.0279
**1.11-1.2**	0	0	1.144	0.0301	1.155	0.0313
**1.21-1.4**	0	0	1.322	0.1808	1.295	0.056
**1.41-1.6**	0	0	1.507	0.0605	1.508	0.0573
**1.61-1.8**	0	0	1.712	0.0637	1.702	0.0596
**1.81-2**	1.899	0.0579	1.897	0.0603	1.897	0.0603
**2.01-2.5**	2.227	0.1438	2.217	0.1462	2.217	0.1462
**2.51-3**	2.752	0.1378	2.735	0.1446	2.735	0.1446

Table S2. Young's elastic moduli of 25 enucleated, 25 nucleated and 60 mix cells derived from indenting cells with conical tip at different locations on the cells, using AFM. Obtained results from all cells were sorted into different elastic ranges and averaged. Standard deviation for each range is also calculated.

Nucleus		
Elastic modulus range (kPa)	Average elastic modulus (kPa)	S.D.
**1.1-3**	2.223	0.5526
**3.1-4.5**	3.819	0.4074
**4.51-6**	5.205	0.4358
**6.1-7.5**	6.706	0.3625
**7.51-9**	8.348	0.4552
**9.1-10.5**	9.749	0.3595
**10.51-12**	11.107	0.4269
**12.1-15**	13.4	0.7756
**15.1-18**	16.558	0.9902
**18.1-21**	19.62	0.0283
**21.1-24**	22.897	0.4105

Table S3. Young's elastic moduli of 60 nuclei derived from AFM measurements applying conical indenter at different locations of the sample. Young's elastic modulus of all indented points for each nucleus were calculated and along with the results from other nuclei sorted into different ranges. Average and standard deviation were obtained for each range.

Bibliography

1. Darling, E. M., Zauscher, S., Block, J. A. & Guilak, F. A thin-layer model for viscoelastic, stress-relaxation testing of cells using atomic force microscopy: do cell properties reflect metastatic potential? *Biophys. J.* **92,** 1784–1791 (2007).

2. Darling, E. M., Topel, M., Zauscher, S., Vail, T. P. & Guilak, F. Viscoelastic properties of human mesenchymally-derived stem cells and primary osteoblasts, chondrocytes, and adipocytes. *J. Biomech.* **41,** 454–464 (2008).

3. Gonzalez-Cruz, R. D., Fonseca, V. C. & Darling, E. M. Cellular mechanical properties reflect the differentiation potential of adipose-derived mesenchymal stem cells. *Proc. Natl. Acad. Sci. U. S. A.* **109,** E1523-9 (2012).

4. Darling, E. M., Zauscher, S. & Guilak, F. Viscoelastic properties of zonal articular chondrocytes measured by atomic force microscopy. *Osteoarthr. Cartil.* **14,** 571–579 (2006).

5. Shin, D. & Athanasiou, K. Cytoindentation for obtaining cell biomechanical properties. *J. Orthop. Res.* **17,** 880–890 (1999).

6. Darling, E. M. *et al.* Mechanical properties and gene expression of chondrocytes on micropatterned substrates following dedifferentiation in monolayer. *Cell. Mol. Bioeng.* **2,** 395–404 (2009).

7. Gonzalez-Cruz, R. D. & Darling, E. M. Adipose-derived stem cell fate is predicted by cellular mechanical properties. *Adipocyte* **2,** 87–91 (2013).

8. Jaasma, M. J., Jackson, W. M. & Keaveny, T. M. Measurement and characterization of whole-cell mechanical behavior. *Ann. Biomed. Eng.* **34,** 748–758 (2006).

9. Zhang, H. & Liu, K.-K. Optical tweezers for single cells. *J. R. Soc. Interface* **5,** 671–690 (2008).

10. Guck, J. *et al.* The optical stretcher: a novel laser tool to micromanipulate cells. *Biophys. J.* **81,** 767–784 (2001).

11. Wang, N., Butler, J. P. & Ingber, D. E. Mechanotransduction across the cell surface and through the cytoskeleton. *Science* **260,** 1124–1127 (1993).

12. Crick, F. H. C. & Hughes, A. F. W. The physical properties of cytoplasm: A study by means of the magnetic particle method Part I. Experimental. *Exp. Cell Res.* **1,** 37–80 (1950).

13. Valberg, P. A. Magnetometry of ingested particles in pulmonary macrophages. *Science* **224,** 513–516 (1984).

14. RAND, R. P. & BURTON, A. C. Mechanical Properties of the Red Cell Membrane. *Biophys. J.* **4,** 115–135 (1964).

15. Lee, L. M. & Liu, A. P. The Application of Micropipette Aspiration in Molecular Mechanics of Single Cells. *J. Nanotechnol. Eng. Med.* **5,** 408011–408016 (2014).

16. Lee, L. M., Lee, J. W., Chase, D., Gebrezgiabhier, D. & Liu, A. P. Development of an advanced microfluidic micropipette aspiration device for single cell mechanics studies. *Biomicrofluidics* **10,** 54105 (2016).

17. Gossett, D. R. *et al.* Hydrodynamic stretching of single cells for large population mechanical phenotyping. *Proc. Natl. Acad. Sci. U. S. A.* **109,** 7630–7635 (2012).

18. Xavier, M. *et al.* Mechanical phenotyping of primary human skeletal stem cells in heterogeneous populations by real-time deformability cytometry. *Integr. Biol. (Camb).* **8,** 616–623 (2016).

19. Koch, M. *et al. Plasmodium falciparum* erythrocyte-binding antigen 175 triggers a biophysical change in the red blood cell that facilitates invasion. *Proc. Natl. Acad. Sci.* **114,** 4225–4230 (2017).

20. Kräter, M. *et al.* Bone marrow niche-mimetics modulate HSPC function via integrin signaling. *Sci. Rep.* **7,** 2549 (2017).

21. Adamo, A. *et al.* Microfluidics-based assessment of cell deformability. *Anal. Chem.* **84,** 6438–6443 (2012).

22. Byun, S. *et al.* Characterizing deformability and surface friction of cancer cells. *Proc. Natl. Acad. Sci. U. S. A.* **110,** 7580–7585 (2013).

23. Chen, J. *et al.* Classification of cell types using a microfluidic device for mechanical and electrical measurement on single cells. *Lab Chip* **11,** 3174–3181 (2011).
